# “Broken Windows”: Frumkin Responds

**Published:** 2005-10

**Authors:** Howard Frumkin

**Affiliations:** Rollins School of Public Health Emory University, Atlanta, Georgia, E-mail: medhf@sph.emory.edu

I thank Meléndez for his careful reading of my editorial and for raising the very reasonable question of whether “broken windows”—an indicator of neighborhood squalor—are causally related to poor health.

Clearly the relationship between features of the built environment—including signs of degradation and outcomes such as behavior and health—is very complex. Many of the causal arrows are probably bidirectional. True clinical trials, which might help disentangle and clarify specific causal pathways, are difficult to carry out, as Meléndez points out. However, at least two interesting studies approximate a trial and are informative.

First, in the mid-1990s, former New York City police commissioner William Bratton implemented a “fixing broken windows” approach—enforcing nuisance laws, cleaning up graffiti, and so on. This approach was credited with a substantial subsequent decrease in street crime (Bratton 1995; Bratton and Knowlner 1998; Kelling and Coles 1996). Second, the Moving to Opportunity trial in the mid-1990s enrolled over 3,000 families in high-poverty neighborhoods of Baltimore, Maryland; Boston, Massachusetts; Chicago, Illinois; Los Angeles, California; and New York, New York. They were randomly assigned to receive housing vouchers usable in low-poverty neighborhoods or to remain where they were. Although the results were variable, families moving to low-poverty neighborhoods did experience improvements in several aspects of physical and mental health (Orr et al. 2003). So while these effects are not simple, there is some evidence that less-chaotic, disordered environments may predict better health.

Perhaps the fundamental issue is that in poor communities, environmental factors and social factors are inextricably intertwined. Our efforts to understand their effects on health, and to improve people’s lives, need to focus on the root causes of both poverty and environmental hazards.
